# Detection of Factors Related to the Development of Osteochondritis Dissecans in Youth Baseball Players Screening

**DOI:** 10.3390/diagnostics13233589

**Published:** 2023-12-03

**Authors:** Shotaro Teruya, Takeshi Ogawa, Hiroki Yamada, Hiromitsu Tsuge, Ryuhei Michinobu, Kazuhiro Ikeda, Yuki Hara, Hiroshi Kamada, Masashi Yamazaki, Yuichi Yoshii

**Affiliations:** 1 Department of Orthopedic Surgery, Institute of Medicine, University of Tsukuba, Tsukuba 305-8571, Ibaraki, Japan; steruya@tsukuba-seikei.jp (S.T.);; 2 Department of Orthopedic Surgery, Kasumigaura Medical Hospital, Tsuchiura 300-8585, Ibaraki, Japan; 3 Department of Orthopedic Surgery, Mito Medical Center, Ibaraki 311-3193, Ibaraki, Japan; 4 Department of Orthopedic Surgery, National Center of Neurology and Psychiatry, Kodaira 187-8551, Tokyo, Japan; 5 Department of Orthopedic Surgery, Tokyo Medical University Ibaraki Medical Center, Ami 300-0395, Ibaraki, Japan

**Keywords:** on-field screening, elbow injury in baseball, osteochondritis dissecans of the humeral capitellum

## Abstract

On-field screening for ‘elbow injury in baseball’, a condition commonly seen in youth baseball players, was conducted over two years on 160 elementary school students in Ibaraki Prefecture, Japan. This on-field screening was conducted in collaboration with the Ibaraki Prefecture High School Baseball Federation. Pitchers, catchers, symptomatic players, and players who had previously experienced elbow pain were given a comprehensive evaluation that included a physical exam and ultrasound. Out of the 135 students who were successfully screened, 10 were diagnosed with osteochondritis dissecans of the humeral capitellum (OCD). Notably, seven among these were asymptomatic. This assessment identified limited range of motion and pain when extending their elbow as significant risk factors for OCD. An attempt at on-field screening for baseball elbow injuries in collaboration with the local baseball federation was introduced. The risk factors for OCD were identified. Considering these factors, more efficient screening will be possible in the next attempt.

## 1. Introduction

Elbow injuries related to baseball are broadly classified into medial, lateral, and posterior types. Osteochondritis dissecans of the humeral capitellum (OCD), a disease representative of the lateral type of elbow injury in baseball, is more common in schoolchildren aged 9–12 years [1,2,3]. If the condition becomes severe, it causes functional impairment to the extent that the player cannot continue to play baseball [4]. Therefore, it is of paramount importance to focus on the early identification and appropriate intervention for OCD in this demographic. Notably, OCD is an affliction that often begins with minimal or no symptoms, a factor that can lead to delayed medical attention and, consequently, a deterioration of the condition by the time treatment is sought. Therefore, proactive early screening initiatives are vital. They hold the potential for enabling conservative treatments that focus on the repair of the lesion, thereby preventing the escalation to more serious stages that could imperil a young player’s ability to continue in the sport.

OCD involves the separation of a section of articular cartilage and the underlying subchondral bone, leading to varying degrees of sclerosis, fragmentation, and resorption [5,6]. OCD affecting the trochlea is less common, accounting for 2.5–7% of all elbow OCD lesions [6,7,8]. While the exact underlying mechanism remains unclear, one hypothesis points to the tenuous blood supply to the posteroinferior aspect of the lateral trochlea [9,10]. Overhead athletes experience repeated impingement when extending their elbows, which can exacerbate blood flow disorders [11]. In recent years, on-field screening for baseball elbow injuries for elementary and junior high school students has been conducted throughout Japan [1,2,3,12,13,14]. Past reports have shown a variety of target age groups, ranging from elementary school students [1,2,12,14] to extending the target to junior high school students and beyond [3,13]. Ultrasonographic examinations are valued in these screenings and have even been conducted at ground level in some cases [2,12]. The size of each screening session varies widely from approximately 100 to 1000 individuals, depending on the region and the environment.

In this context, it has been noted that until now, no screenings for elbow injury in baseball have been conducted in our region (Ibaraki Prefecture). This was due to a lack of know-how and human resources. On the other hand, upon request from the Prefectural High School Baseball Federation, post-game medical checks began to be conducted. Through these checks, it was discovered that a significant number of elementary and junior high school students have experienced throwing-related issues. In Ibaraki Prefecture, elbow-injury-in-baseball on-field screening for elementary school students has been conducted at the same time as youth baseball lessons organized by the Ibaraki High School Baseball Federation since 2016. Considering the number of players and the fact that screenings have not been conducted so far, it is conceivable that initiating screenings in these unscreened regions could potentially detect previously unrecognized cases. Furthermore, conducting elbow injury screenings could raise community awareness about this issue.

This study aimed to evaluate the effectiveness of our prefecture’s approach to diagnosing and managing elbow injury in baseball among school-aged athletes and to consider more efficient screening methods with regard to detecting factors related to the development of OCD.

## 2. Materials and Methods

### 2.1. Participants

**Figure 1:**
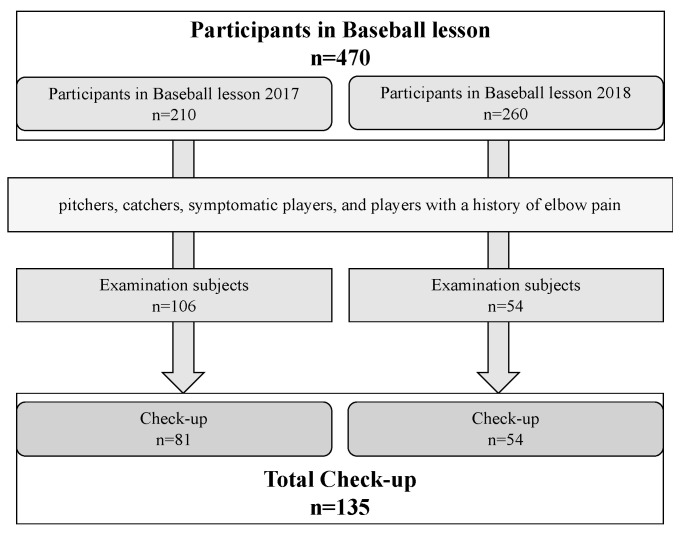
In total, 135 athletes were screened in two years.

The study involved 160 subjects, including pitchers, catchers, players showing symptoms of elbow pain, and players with a previous history of elbow pain from a total of 470 baseball players of elementary school students (50 teams in total) who participated in youth baseball lessons organized by the prefectural high school baseball federation over a two-year period from 2017 to 2018 (Figure 1). None of the players had a medical check-up two years in a row.

### 2.2. Field Screening

**Figure 2:**
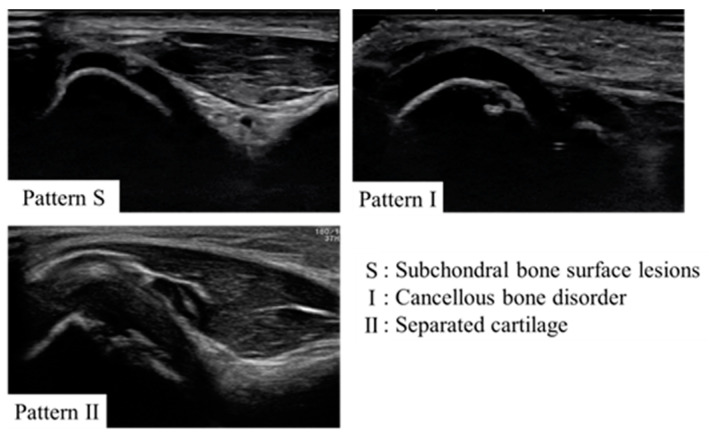
Characteristic ultrasound images of OCD extracted during the current screening are shown. This study followed Okada’s pattern classification.

First, the subjects were given a medical questionnaire in which they were asked to provide their name, team, position, and history of elbow pain. Physiotherapists and trainers then assessed the elbow for pain, limited motion, tenderness of the medial epicondyle, tenderness of the humeroulnar or humeroradial joint, tenderness of the olecranon, and instability with valgus stress tests at 30 degrees, 60 degrees, and 90 degrees of elbow flexion for both the throwing and non-throwing sides. The elbow joint was then examined by a physician using ultrasound to confirm the presence of OCD; the Okada’s pattern classification was used to assess OCD [15] (Figure 2). The ultrasound examination focused solely on the lateral side of the elbow, checking both the short axis and the long axis. The equipment used for the ultrasound examination was a portable ultrasonography and an 11-MHz linear array transducer (SONIMAGE MX1, KONICA MINOLTA JAPAN Inc., Tokyo, Japan). The ultrasound examinations were performed by orthopedic surgeons with 3–10 years of experience. For the final analysis, the ultrasound images were interpreted by multiple physicians, including a specialist with over 20 years of experience in orthopedics. In this study, following the pattern classification, those with marginal irregularities were extracted, and the pattern S, which is typically not counted as OCD in a single examination, was also included as OCD for the purposes of this screening.

Players exhibiting severe pain, limited motion, or abnormal ultrasound findings were flagged for further investigation. The medical information form was prepared for a nearby medical facility, and they were instructed to undergo a secondary screening. In this study, if there were physical findings such as elbow pain, immediate aftercare instructions were provided on the spot. If the ultrasound examination indicated the presence of OCD, or if there was a suspicion of this condition, patients were advised to undergo a follow-up examination.

**Figure 3:**
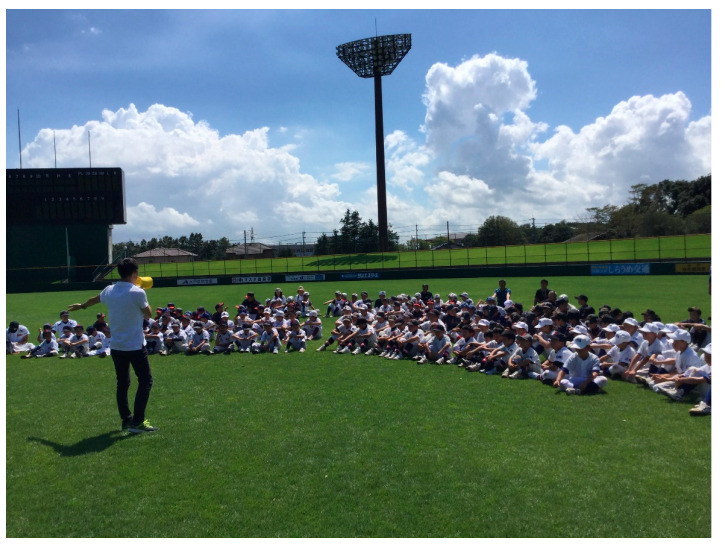
A scene at the lecture on elbow injuries related to baseball.

Additionally, an orthopedic surgeon lectured the participants and their parents about baseball-related elbow injuries (Figure 3). In the lecture, we primarily discussed medial and lateral elbow injuries in baseball. Furthermore, it was emphasized that screening tests are of utmost importance in the case of lateral elbow injuries which can lead to long-term absence from play. Early detection and prompt therapeutic intervention were highlighted as crucial factors.

### 2.3. Statistical Analysis

We analyzed the association between the physical findings and the occurrence of OCD, using the chi-square test. Individuals in the study were stratified into two distinct groups for analytical clarity. The subjects who did not exhibit OCD were labeled as Group N, while those diagnosed with OCD were categorized as Group O. Following the chi-square analysis, we proceeded with logistic regression analysis on the variables that exhibited a statistically significant difference.

## 3. Results

There were 160 subjects eligible for screening, of whom 135 completed the screening. The subjects were distributed across grades as follows: 43 were 12 years old, 47 were 11 years old, 21 were 10 years old, 17 were 9 years old, and 7 were 8 years old; the grade was unknown for four subjects. In terms of positions, 67 were pitchers, 28 were catchers, 23 were infielders, 15 were outfielders, and two were unknown positions. Of the total subjects who completed the screening, 31 warranted further evaluation. This determination was based on a combination of physical examination results and ultrasound findings, emphasizing the importance of a multi-faceted approach to screening. Within this subgroup necessitating additional investigation, 10 subjects, which constituted 7.4% of those who were screened, were diagnosed with OCD. The distribution by position was five pitchers, two catchers, two outfielders, and one position unknown. Within this subset of players, seven were identified as being asymptomatic. There was one symptomatic case in Pattern S (Case 4) and two symptomatic cases in Pattern 2 (Case 9, 10).

**Figure 4:**
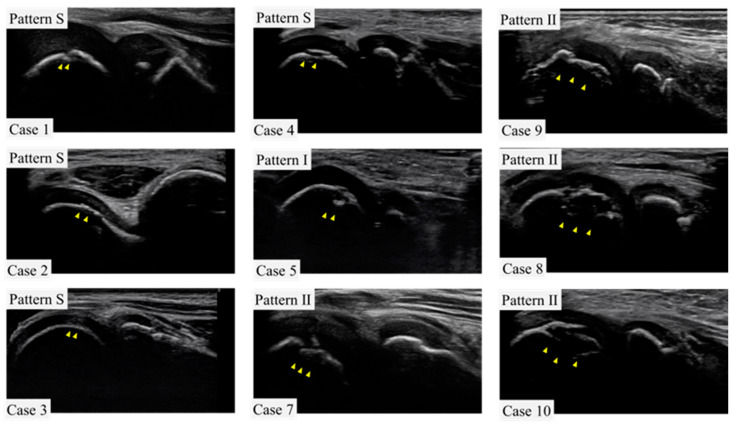
Ultrasound images of the lateral elbow for each case in this study. For one case (case 6), only the record was available, and the image was not preserved. Arrowheads indicate sites of OCD.

Those with complaints of elbow pain and joint tenderness were considered symptomatic. Physical examination revealed pain in 19 players, including 2 who were part of Group O. There was limited extension observed in eight players, half of whom belonged to Group O. Pain during extension was present in five players, with two being from Group O. Limited flexion was found in eight players, with two in Group O; pain on flexion was noted in four players, with none in Group O. Tenderness at the medial epicondyle was evident in 27 players, with 3 in Group O, while tenderness at the humeroradial joint was reported in 10 players, including 1 in Group O. Furthermore, tenderness at the olecranon was found in five players, with one from Group O. The valgus stress test was positive at 30 degrees of elbow flexion in 13 players, with 1 in Group O; at 60 degrees, it was positive in 16 players, with none in Group O; and at 90 degrees, it was positive in 13 patients, with none in Group O (Table 1). Of those diagnosed with OCD, five exhibited pattern S, one had pattern 1, and four presented with pattern 2 (Figure 4).

In our investigation, we employed the chi-squared test to assess the correlation between various physical signs and the incidence of osteochondritis dissecans (OCD). This statistical test indicated a notable correlation between the manifestation of OCD and specific physical symptoms: notably, individuals with OCD experienced significant pain upon elbow extension (*p* = 0.005), and there was a marked restriction in their range of motion during elbow extension (*p* < 0.001), highlighting these as potential indicators of the condition. Subsequently, we conducted a logistic regression analysis with the goal of establishing the presence of OCD as the dependent variable. In this model, we incorporated the physical findings that exhibited statistically significant differences in the chi-squared test as the independent variables. Limited range of motion in elbow extension (OR, 19.42; CI, 3.61–104.60; *p* < 0.001) and pain in elbow extension (OR, 9.38; CI, 1.03–85.67; *p* = 0.047) were identified as significant risk factors for OCD (Table 2). 

## 4. Discussion

There are approximately 200 youth baseball teams in Ibaraki Prefecture. Each year, approximately 25 teams from Ibaraki Prefecture participate in the screening, which is a small fraction of the nearly 200 youth baseball teams in the region. By examining a specific subset of positions within these teams, it is estimated that the participants in this screening represent about 3–5% of the total youth baseball population in the prefecture. One hundred and thirty-five patients were examined for on-field screening of elbow injuries related to baseball. It was found that ten of the subjects had OCD, seven of whom were asymptomatic. Twenty-five of the subjects did not join the screening because of team or family reasons. 

In the youth baseball, the role of the pitcher is frequently assumed by those players who possess a rich background in the sport and can deliver pitches at high velocities. This group, as a result, is subject to substantial chronic elbow stress. The notably high occurrence rate of osteochondritis dissecans (OCD) detected in our study may be largely ascribed to our focus on central team players, namely pitchers and catchers, as well as those individuals reporting elbow discomfort. Existing research reports that the prevalence of elbow pain among young pitchers is roughly 26% [16]. It has been postulated that pitchers, due to the significant physical demands placed on their upper limbs during play, suffer from an increased rate of elbow pain in comparison to their counterparts in other positions [17,18,19,20]. With the objective of improving the effectiveness and precision of our screening process, we strategically confined our examination to pitchers, catchers, and those players with a documented history of elbow issues. The duration necessary for conducting this examination was approximately one and a half hours. Should we have extended this screening to encompass all members of the baseball teams, we estimate it would necessitate about four and a half hours—a threefold increase in time. Despite the overarching goal of a screening being to evaluate as comprehensive a player population as possible, we found that the examination proceeded efficiently, albeit within the constraints posed by limited staff and time availability. In a comparative light, our findings align with or diverge from other regional or international studies in intriguing ways. We selected the specific sampling method employed to target pitchers, catchers, and those experiencing elbow pain. Although this method efficiently detects OCD cases, it may have introduced a selection bias, warranting a discussion on the potential skewness in the data representation. It also raises the question of how universal screening across all player positions could reveal OCD prevalence more broadly.

Previous reports have shown that the prevalence of OCD in baseball elbow screening ranges from 1.2% to 3.4% [3,12,21]. In our study, the proportion of subjects who were deemed to require a secondary, more detailed screening was found to be 23.0%, which is substantially higher than previously reported. Moreover, a significant 7.4% of all the subjects examined were conclusively diagnosed with OCD by means of ultrasound evaluation. Both of these percentages represent high rates, which can be attributed to the specific selection of subjects for the study. 

This investigation has determined that a limited range of motion during elbow extension and pain experienced upon extending the elbow are significant risk factors associated with OCD. Interestingly, there have been scant previous reports that make a connection between the specific pain during elbow extension and the onset of OCD, which positions our findings as potentially groundbreaking. By bringing into focus the symptom of elbow extension pain—a facet that has not been extensively explored in prior research—we can potentially broaden the scope of candidates eligible for screening examinations. Should further research consolidate the link between elbow extension pain and OCD, this symptom could become a crucial criterion for advising against continued pitching. This advancement would not only contribute to medical guidelines but could also inform immediate, on-the-spot decisions to cease pitching activities at the field level, enhancing preventive measures and safeguarding the health of players.

Prevention, timely detection, and early intervention of elbow injuries in school-aged athletes are crucial to mitigating the onset of chronic elbow pain later in life. Baseball-related elbow injuries, especially osteochondritis dissecans (OCD), often present without symptoms in their initial stages. However, more severe cases may necessitate surgical intervention, raising concerns about potential lasting functional impairments. Consequently, conducting regular medical checkups focused on the elbow is essential for pre-empting injuries related to pitching. In the context of youth baseball, the early identification of OCD can pave the way for recovery through rest and non-invasive treatments. In contrast, delays in detection can lead to extended periods of discomfort and substantial challenges in maintaining pitching performance. Moreover, while a player might not experience pain during their youth baseball activities, it is possible that sudden pain could manifest as they progress in the sport. Under such circumstances, opportunities for repair through conservative treatment may diminish, often making surgical solutions a more common recourse. Therefore, implementing consistent screening protocols and promptly addressing any signs of discomfort are vital. These measures can facilitate the early detection and management of elbow injuries, thereby safeguarding the player’s long-term engagement and performance in baseball.

In recent years, the scope and methods of elbow injury screenings in baseball have varied across regions. Iwame and Matsuura et al. have successfully promoted the importance of elbow screening over the years. They have now been able to secure a large population by holding the examinations in conjunction with youth regional baseball tournaments [22,23]. Kida et al. carry out baseball screenings for youth aged 12–18 during the off-season, synchronizing these examinations with regional baseball training camps to effectively capture a substantial participant base. Additionally, they conducted the program at the request of the Rubber Baseball Association, the Junior High School Baseball Federation, and the High School Baseball Federation. Over the course of a year, approximately 2500 participants, ranging from elementary to high school students, took part in these examinations [3]. They have successfully conducted large-scale elbow screenings by synchronizing the examinations with events such as regional tournaments and training camps, ensuring the participation of a broad range of young players. Sakata et al. conducted a pre-season medical check-up on 593 youth players aged 6–12 by the same examiner. Their findings indicated that pitchers had a significantly higher incidence of medial elbow injuries compared to other positions [18]. Tajika et al. screened 164 baseball players from regional youth baseball teams. They studied the occurrence of pain pre- and post-season, examining its correlation with physical changes and other clinical observations. Their findings revealed a significant relationship between pre-season body weight and the onset of elbow pain [24]. Otoshi et al. carried out baseball screenings post-season and studied the relationship between elbow pain and the off-season’s length. Their findings indicated that a prolonged off-season corresponded to reduced occurrences of medial elbow pain. Yet, they observed no association between the off-season duration and the onset of OCD [1]. They have shown through pre- and post-season screenings that there is a correlation between the players’ physical changes, the length of the off-season, and clinical symptoms. This information could be valuable for choosing future treatment and prevention strategies. Takata et al. conducted a screening of 1045 players with current or past elbow pain. There was a notable difference in the detection of OCD between those who received an ultrasound examination and those who did not, with a higher detection rate in the former group. Furthermore, the ultrasound group also showed a higher follow-up examination attendance, underscoring the efficacy of ultrasound in such screenings [14]. Harada et al. conducted baseball screenings using ultrasound examinations and found that, out of 153 players, 35 presented with medial abnormalities detected by the ultrasound [12]. They demonstrated that the use of ultrasound allowed for more accurate detection of elbow abnormalities and promoted increased attendance at follow-up examinations. Outside of Japan, Holt et al. examined both elbows using MRI before and after the season to track any changes in condition. Their findings suggested that continuous play throughout the year significantly exacerbated MRI-detectable abnormalities [25]. However, given the use of MRI, this study goes beyond a simple screening examination. While baseball elbow screenings occur in many places, their scope and setting differ regionally. Similarly, proactive prevention approaches differ. When introducing screenings to previously underserved areas, challenges arise, such as selecting an appropriate venue, determining necessary staffing, and ensuring the availability of equipment like ultrasound machines. Given these screenings’ nature, it is vital to swiftly and effectively assess many players to fulfill the screening’s intended purpose.

In Ibaraki Prefecture, baseball lessons are held twice a year for members of the high school baseball teams. It was thought that the number of participants could be increased if the elbow screenings were held at the same time. The prefectural high school baseball federation readily agreed to the event because some players are unable to pitch in high school due to injuries sustained during their youth baseball days, and the event may lead to an increase in the future population of high school baseball players. Many of the youth baseball players also aspire to play in the Koshien National High School Baseball Championships. Therefore, we believed that the participation rate of screening would increase if they were encouraged by high school baseball players and coaches. As the new project, Ibaraki Prefecture also introduced elbow-injury-in-baseball on-field screening. It was not an easy task to raise awareness in areas where there had been no elbow injury screening in the past. We believe that holding the on-field screening at the same time as baseball lessons sponsored by the High School Baseball Federation helped to raise awareness. Unlike other sports, baseball has many federations. While soccer, athletics and other sports have their own federations under a larger federation, baseball, especially junior high school baseball, has many individual federations. This situation makes it difficult to hold health screenings. Therefore, we thought about collaborating with the High School Baseball Federation, to which most players belong if they continue to play baseball until their high school age. High school baseball is still very popular in Japan, and the prefectural high school baseball federations believe that popularization of preventive medicine will lead to the prevention of injuries. In addition, we also conducted on-field screening using mobile MRI [26]. When combined with mobile MRI, it may be possible to make a definitive diagnosis at the time of screening.

Limitations of this study include limited human resources and time. As this was a new project, the number of participants on the medical side was small and only pitchers, catchers, symptomatic players, and players with a history of elbow pain were included. Physical examination findings and ultrasound were used to diagnose OCD in this study. The study did not extend to the receipt and results of secondary medical examinations and did not reflect the results of X-rays and MRIs, which are commonly performed at secondary screening. In the future, we can increase the number of participants through the following: (1) by increasing the number of medical staff participants, and (2) arranging alternative dates for teams who are aware of the existence of OCD but are unable to participate due to tournaments, matches, and other reasons. In addition, it is necessary to increase cooperation with secondary health screening facilities. In this study, ultrasound examinations were performed by multiple examiners. While it was possible to evaluate OCD of the lateral epicondyle of the elbow, it should be noted that the ability to capture images may vary depending on the skill level of the examiner. Furthermore, while we used the Pattern classification, it should be noted that our study was designed as a screening procedure. At present, we have not implemented changes in the treatment methods based on different Patterns. Future longitudinal studies with larger datasets might enable more definitive treatment decisions. It would also be desirable to evaluate medial epicondylitis of the elbow simultaneously and require even advanced proficiency in ultrasound examination techniques. There is a need for standardization in the methods of ultrasound imaging and evaluation across different examiners. Efforts have also been made to standardize the ultrasound examination method and improve the detection rate for medial baseball elbow injuries [27]. Moving forward, there will be a continuous search for more effective examination methods in elbow screenings.

The long-term implications of early OCD detection and treatment go beyond merely the physical realm. There is a socio-economic dimension considering the cost of treatment in advanced stages and a psychological facet given the mental strain on young players and their families. The value proposition of early interventions transcends the immediate health benefits and delves into the holistic wellbeing of the affected individuals and the community. Moreover, the success of this initiative in Ibaraki Prefecture beckons the exploration of it across other prefectures or even on a national scale. Follow-up studies, coupled with educational campaigns targeting coaches, players, and parents, can propagate the essence of early detection and preventive measures. The findings advocate for a structured framework within youth sports safety protocols, emphasizing regular health screenings and early interventions. The potential to translate these findings into actionable policies could significantly advance the overarching goal of fostering a safe and health-conscious sporting environment.

## 5. Conclusions

We have reported an attempt to conduct on-field screening of elbow injuries in the areas where elbow joint examinations have not been performed previously. Although we were able to introduce the system smoothly by cooperating with the local Higher-Level Baseball Federation, our efforts have shown that there is room for improvement. The prevalence of OCD was as high as 7.4% in the examination. The incidence was significantly higher than previous studies in other regions. This high rate may suggest that our on-field screening was efficient at catching cases that might have otherwise gone unnoticed. However, it may also be due to the limited population screened, indicating a need for broader and more frequent screening events. In addition, it is necessary to improve the efficiency of examinations. It was found that limited range of elbow extension and pain on elbow extension may be related to the onset of OCD. These findings not only align with existing medical knowledge but also pave the way for focused preventive measures. Collaborative efforts, integrating the expertise of medical professionals, the support of baseball federations, and the advancement in diagnostic technologies, form the cornerstone of evolving a sustainable and impactful preventive healthcare model within the youth baseball echo-screening system.

## Figures and Tables

**Table 1:** Results of physical examinations.

	Group N	Group O	*p* Value
Total number of players	125	10	
Throwing pain	17	2	0.58
Limited ROM in extension	4	4	<0.001
Extension pain	3	2	0.005
Limited ROM in flexion	4	0	0.57
Flexion pain	6	2	0.0501
Tenderness of medial epicondyle	24	3	0.41
Tenderness of brachial joint	9	1	0.74
Tenderness of olecranon	4	1	0.27
30° valgus stress test	12	1	0.97
60° valgus stress test	16	0	0.23
90° valgus stress test	13	0	0.28

**Table 2:** Risk factors for developing OCD by logistic regression analysis.

	OR (95% CI)	*p* Value
Extension pain	19.42 (3.61–104.60)	<0.001
Limited ROM in extension	9.38 (1.03–85.67)	0.047

## Data Availability

Data are contained within the article.
